# Comparative Transcriptome Analysis of Gene Expression Between Female and Monoecious *Spinacia oleracea* L.

**DOI:** 10.3390/genes16010024

**Published:** 2024-12-27

**Authors:** Yingjie Zhao, Zhiyuan Liu, Hongbing She, Zhaosheng Xu, Helong Zhang, Shaowen Zheng, Wei Qian

**Affiliations:** 1 College of Horticulture, Shanxi Agricultural University, Taigu, Jinzhong 030801, China; 15511727779@163.com; 2 State Key Laboratory of Vegetable Biobreeding, Institute of Vegetables and Flowers, Chinese Academy of Agricultural Sciences, Beijing 100081, China; liuzhiyuan01@caas.cn (Z.L.); shehongbing@caas.cn (H.S.); xuzhaosheng@caas.cn (Z.X.); zhanghelong@caas.cn (H.Z.)

**Keywords:** spinach, monoecious, RNA-seq, differentially expressed genes (DEGs)

## Abstract

Background: Spinach (*Spinacia oleracea* L.) is an important leafy vegetable with dioecious and occasional monoecious plants. Monoecious lines are more suitable for hybrid production than dioecious lines due to their extended flowering period. However, genetic research on the sex determination of monoecism remains limited. Methods: In this study, RNA-seq analysis of monoecious and female spinach plants was performed at two distinct flowering stages. In total, we identified 4586 differentially expressed genes (DEGs), which were primarily involved in biological processes such as hormone signaling, cell wall biosynthesis, photosynthesis, and flower development, based on Gene Ontology (GO) enrichment analysis and Kyoto Encyclopedia of Genes and Genomes (KEGG) pathway analysis. Results: Among these DEGs, 354 transcription factors, including 27 genes associated with the ABCDE gene, were discovered. Furthermore, a co-expression gene regulatory network was built, identifying nine key genes that play important roles in regulating sex differentiation between female and monoecious plants. Conclusions: Our findings provide crucial molecular insights into the mechanisms of monoecism in spinach and offer a scientific basis for future spinach breeding.

## 1. Introduction

Sex determination and expression in plants is a complex biological process influenced by a combination of genetic, environmental, physiological, biological, and anthropogenic factors. Male plants of dioecious spinach varieties exhibit rapid senescence and a short flowering period, whereas monoecious plants exhibit slower senescence and longer flowering periods and are capable of self-fertilization for seed production. Therefore, the study of sex-related genes in monoecious spinach is critical for breeding. Sex differentiation in plants is governed by both intrinsic factors, such as endogenous hormones, and extrinsic factors, such as environmental conditions. These factors collectively govern and regulate plant sex determination [1]. Studies have shown that plant sex is determined by sex chromosomes (XY system), with the sex-determining gene on the Y chromosome playing a pivotal role [2,3]. Most species in the plant kingdom are monoecious (with bisexual flowers, bearing bisexual flowers, where both pistils and stamens are located in the same flower, or unisexual flowers, where a flower contains only pistils or stamens), and approximately 6% of angiosperms are dioecious, which is characterized by separate male and female flowers located on different plants [4]. The sex-determination system varies significantly across breeding systems. In dioecious species, sex determination is typically controlled by nuclear genes located on sex chromosomes. Only a subset of species exhibits heteromorphic sex chromosomes, similar to most animals. Dioecy in plants may have evolved from monoecy, with sex chromosomes arising from a pair of autosomes [5].

Spinach (*Spinacia oleracea* L.) is an annual and biennial herbaceous plant belonging to the Amaranthaceae family. It is widely cultivated and highly valued worldwide as a nutritious leafy vegetable rich in vitamins (such as vitamin C and vitamin K) and minerals (including iron and calcium). Spinach can be cultivated year-round and serves as an important edible green vegetable during the spring, autumn, and winter seasons [6]. Although most spinach plants are dioecious, monoecious individuals are occasionally observed [7]. Sex determination in dioecious spinach is governed by a pair of *X/Y* alleles located on the largest chromosome [8]. Spinach exhibits an *XY* sex-determination system, with no significant size difference between the *X* and *Y* chromosomes [7]. Spinach exhibits three types of sex expression: male, female, and monoecious. Spinach flowers are unisexual. Most spinach varieties are dioecious, with distinct male and female plants typically showing a 1:1 segregation ratio. In spinach, sex determination is controlled by sex-determining genes on sex chromosomes. Previous studies have shown that *X/Y* genes control dioecy in spinach and the *X^m^* gene governs monoecy. The *Y* chromosome is dominant over both *X* and *X^m^*, and *X^m^* is dominant over *X* [9]. Therefore, the spinach genotypes are as follows: male plants are *XY* or *X^m^Y*, monoecious plants are *X^m^X^m^* or *X^m^X*, and female plants are *XX*. Sex determination and differentiation are crucial biological processes in unisexual flower development. Spinach serves as a model plant for studying plant sex determination and differentiation mechanisms [10]. Despite these advances, research on the *X^m^* gene responsible for monoecy in spinach is still limited, and positional cloning has not yet been achieved. Furthermore, the molecular mechanisms underlying the sex determination network in monoecious spinach remain poorly understood, significantly hindering the breeding efficiency of spinach.

In higher plants, plant hormones, such as auxin (IAA), ethylene, gibberellin (GA), and abscisic acid, play multiple roles in plant growth and development [1,11,12], with many exhibiting pleiotropic effects. IAA induces female flower development in cucumber, lemon, and hemp. GA promotes male flower development in cucumber, melon, asparagus, and hemp but induces female flowers in maize. These hormones also influence sex differentiation in monoecious and dioecious species. Collectively, these findings show that no single hormone has a universal effect on sex determination in monoecious and dioecious plants, indicating that each species has its own hormonally regulated mechanism for sex expression. GA is a major plant hormone that participates in normal plant growth and development and influences sex expression in many plant species [13,14,15,16,17]. Among plant hormones, GA plays a key role in regulating flowering in the model plant *Arabidopsis thaliana*. Although over 100 GAs have been identified in plants, only a few are considered biologically active, including GA1, GA3, GA4, and GA7 [18]. GAs were initially recognized for their effects on stem elongation, with exogenous GA3 application reversing the dwarf phenotypes of *Pisum sativum* [19] and *Zea mays* mutants [20], enabling them to reach heights comparable to mature plants. GAs are essential at multiple stages of plant growth and development, including seed germination, floral induction, leaf elongation, and fruit growth. GAs have also been shown to promote male flower formation in several species, including spinach [21], hemp [22,23], and cucumber. Although GA typically promotes male floral development, it is not universal across all species. For example, exogenous GA3 application in *Z. mays* results in feminization of the terminal inflorescence [24]. Exogenous GA treatment of spinach leads to the transformation of 78% of pistils into stamens [21]. GA3 has been shown to enhance the masculinizing effect on individual female spinach plants, resulting in complete conversion and functional stamens [25].

In many plants, sex determination is controlled by specific genes, and the expression of these genes is often regulated by transcription factors (TFs). For example, in some plants, TFs control the plant’s sex expression by activating or repressing sex-determining gene transcription. The MADS-box gene family plays a pivotal role in numerous stages of the plant developmental cycle, including floral organogenesis, fruit development, and gametophyte formation [26,27]. These genes are also involved in biological regulatory processes in plants, such as circadian rhythm regulation, metabolic regulation, and floral transition [26,28,29]. Some MADS-box genes in angiosperms exhibit homologous functions. Although not all MADS-box genes are homologous, they can serve as key meristem identity regulators. CRC genes from the YABBY family influence carpel development and affect male and female flower formation, playing a pivotal role in sex determination in Cucurbitaceae species [30]. Proposed in 1991 to explain the role of homologous genes in floral organ identity, the ABC model posits that each organ within a floral whorl is determined by the interaction of three distinct organ identity genes [31,32,33,34]. This model was later expanded to the ABCDE model [35,36].

In contemporary plant research, the utilization of transcriptomic data represents a prevalent and popular approach to identify genes associated with sex determination and flowering. Using transcriptomic data from different periods of pepper floral organ development and incorporating the ABCDE model of floral development, 17 ABCDE model candidate genes were identified in pepper by Tang et al. [37]. The transcriptomes of different sexes were analyzed in papaya, and the expression of 40 plant-conserved miRNAs and 14 papaya-specific miRNAs was detected in 1 or 2 normal flowers and pistils of monoecious, inverted flowers by Lin et al. [38]. These results suggest that male-to-female sex reversal may be caused by the silencing of androgynous inhibitory sex functions through epigenetic modification in the sex-determination pathway.

AP1 (Apetala1) regulates sepal and petal development and is expressed during early floral development, playing a key role in flower formation [36]. The PI gene plays a critical role in flower development, particularly in the formation of the second and third whorls of floral organs (i.e., the stamen and pistil). PI expression influences petal and stamen development [39]. AG (Agamous) is essential for stamen and pistil development. AG is a key gene in floral development, and its absence leads to abnormal reproductive organ development [40]. SQUA (Squamosa) participates in stamen and pistil formation in some plant species and affects floral morphology [41]. SEP1 is a member of the SEPALLATA gene family and regulates floral organ development. SEP1 plays an important role in the formation of floral organs, such as petals, stamens, and pistils [42]. Suppressor of Overexpression of Constans 1 (SOC1) integrates environmental signals and activates the flowering process. SOC1 is typically a part of plants’ biological clock and responds to environmental cues, such as photoperiod and temperature, to regulate flowering [43].

In this study, RNA sequencing (RNA-seq) was conducted in female and monoecious plants at two flower stages. We conducted a co-expression network analysis and identified many TF genes. These findings provide novel genetic reservoirs for further pinpointing the regulatory mechanism underlying monoecism.

## 2. Materials and Methods

### 2.1. Plant Materials

A monoecious spinach line (Sp139) and a female spinach line (Sp140) were grown in a nursery tray in a greenhouse at the experiment station of the Institute of Vegetables and Flowers, Chinese Academy of Agricultural Sciences, Beijing, China, in December 2023. These plants were transplanted to the field at the 2−3 leaf stage. Flowers were harvested in April 2024 and used for RNA preparation and RNA-seq analysis. However, due to an insufficient number of representative female plants at the time, only two biological replicates were collected. Samples were collected on the first day of flowering (T1) and the eighth day of flowering (T2). All samples were immediately flash frozen in liquid nitrogen and stored at −80 °C for subsequent analysis.

### 2.2. RNA Extraction and Sequencing

RNA was extracted using a magnetic bead-based method. The concentration and purity of the extracted RNA were assessed using a NanoDrop 2000 and 8000 spectrophotometer (Thermo Fisher Scientific, Waltham, MA, USA). RNA integrity was evaluated using an Agilent 2100 Bioanalyzer (Agilent Technologies, Santa Clara, CA, USA), and RNA was diluted to an appropriate concentration prior to assessment. RNA concentration should typically be between 100 ng/μL and 1 μg/μL. After successful quality control, 1–3 µg of total RNA from each sample was used as the input for RNA-seq library construction. mRNA was subsequently enriched and fragmented into short sequences. First-strand cDNA was synthesized using mRNA as a template, which was followed by second-strand cDNA synthesis. cDNA was then purified, and the resulting products underwent a series of procedures, including end repair, adapter ligation, A-tailing, and PCR amplification, to produce a cDNA library for sequencing.

The constructed cDNA library was quality-checked. After passing quality control, the cDNA library was sequenced on the NovaSeq 6000 S4 platform using paired-end sequencing (150 bp). Raw reads were processed using bioinformatics pipeline tools on the BMKCloud platform (www.biocloud.net, accessed 29 September 2024).

### 2.3. qRT-PCR Analysis and Statistical Analysis

RNA was extracted from each sample and reverse-transcribed into cDNA using HiScript III All-in-One RT SuperMix Perfect (Vazyme, Nanjing, China). qRT-PCR was performed using Taq Pro Universal SYBR qPCR Master Mix (Vazyme, Nanjing, China) on an ABI real-time PCR system (ABI Q1 system). Primers were designed using Primer3 software (v2.6.0, https://primer3.org/, accessed on 10 October 2024; Supplementary Table S2). Gene expression data were analyzed using the 2^−∆∆Ct^ method with actin as an internal reference gene. One-way analysis of variance (ANOVA) was conducted using SPSS v26.0 (SPSS software, Chicago, IL, USA), and Duncan’s multiple range test was used, with the significance level set at *p* < 0.05.

### 2.4. RNA-seq Analysis

The raw sequencing data were subjected to quality control and filtered using fastp (v0.23.3) [44]. HISAT2 (v2.8.2) was used to map clean reads on the spinach reference genome (Monoe_Viroflay) [37]. We used featureCounts (v2.0.1) to calculate the gene expression levels [38]. The expression data were then normalized. Genes with an adjusted *p*-value < 0.01 and fold change ≥2 found by DESeq2 were assigned as differentially expressed.

### 2.5. GO and KEGG Pathway Enrichment Analyses

To identify Gene Ontology (GO) terms that were significantly enriched compared with the entire genome background, enrichment analysis for biological processes, molecular functions, and cellular components was performed on the DEG sets of each group using the hypergeometric test in ClusterProfiler. The Kyoto Encyclopedia of Genes and Genomes (KEGG) pathway enrichment results were visualized using graphical representations generated by ClusterProfiler software (v4.4.4) [45].

### 2.6. Analysis of Co-Expression Trends and Co-Expression Networks

Co-expression trends were analyzed for 10 samples using the K-means method in the Python (v3.12.4) package [46].

### 2.7. Transcription Factor Prediction

The DEGs were subjected to TF prediction using the PlantTFDB database (https://planttfdb.gao-lab.org/, accessed on 10 October 2024) and further analyzed in conjunction with the ABCDE model of floral development to identify relevant genes.

## 3. Results

### 3.1. RNA Sequencing

**Figure 1:**
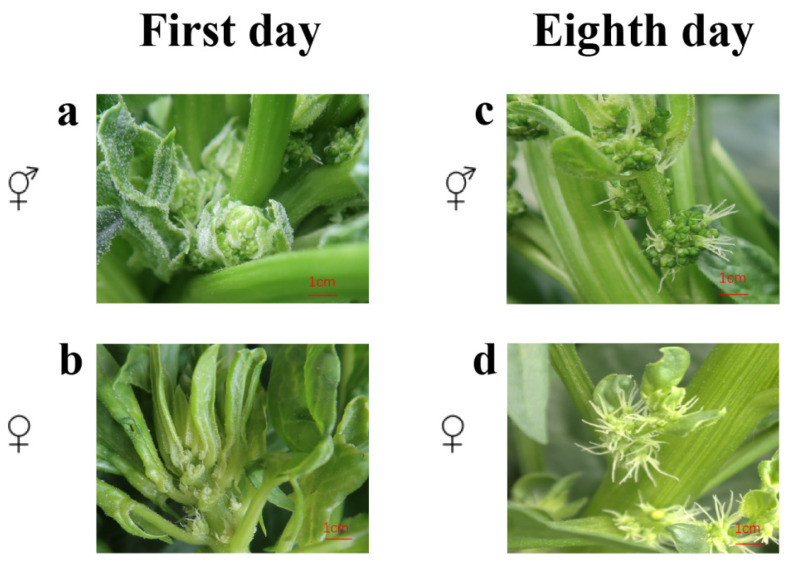
Sampling time periods of Sp139 and Sp140. (**a**) First day of flowering in Sp139; (**b**) first day of flowering in Sp140; (**c**) eighth day of flowering in Sp139; and (**d**) eighth day of flowering in Sp140.

**Figure 2:**
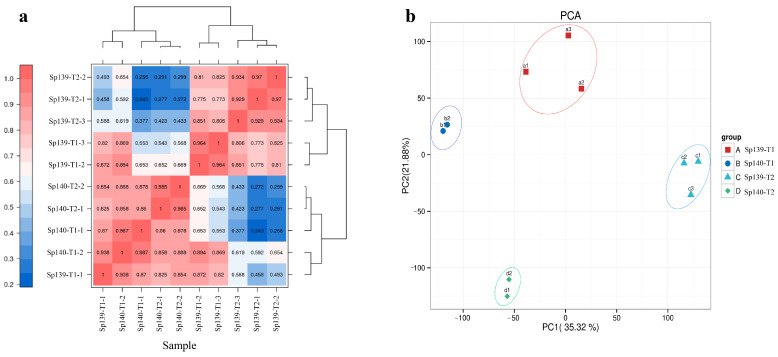
Repeated correlation assessment and PCA. (**a**) Pearson correlation coefficients for comparisons among all samples and (**b**) PCA based on all expressed genes, showing three distinct sample groups.

RNA-seq of the cDNA libraries were constructed from 10 spinach flowers of 2 lines (Sp139 and Sp140) at 2 stages (T1 and T2), generating 65.16 Gb of clean data, with >6.00 Gb for each sample (Figure 1). All clean reads were aligned to the Monoe–Viroflay spinach reference genome. The overall alignment rates ranged from 88.89% to 93.75% (Supplementary Table S1). Based on the selected reference genome, we utilized the StringTie software (v2.2.1) to assemble mapped RNA-seq reads. The resulting assembled transcripts were compared against the existing genomic annotation to identify previously unannotated transcriptional regions, thereby discovering novel transcripts and genes. To ensure the reliability of our findings, sequences encoding peptides shorter than 50 amino acid residues or containing only a single exon were excluded from further analysis. This rigorous filtering process resulted in the identification of 3152 novel genes, significantly enhancing the completeness and accuracy of the original genome annotation. To assess the accuracy and reproducibility, we determined the Pearson correlation coefficient between samples within the group, and the value ranged from 0.82 to 0.98, indicating high reproducibility between samples (Figure 2a). These results demonstrate the high quality of the transcriptome data, indicating its suitability for further analysis. Principal component analysis (PCA) was conducted to reduce the dimensionality of multiple variables into a few independent components (principal components). The PCA plot showed a clear separation between groups, with significant differences between samples (Figure 2b).

### 3.2. Identification of Differentially Expressed Genes (DEGs) Between Monoecious and Female Individuals

**Figure 3:**
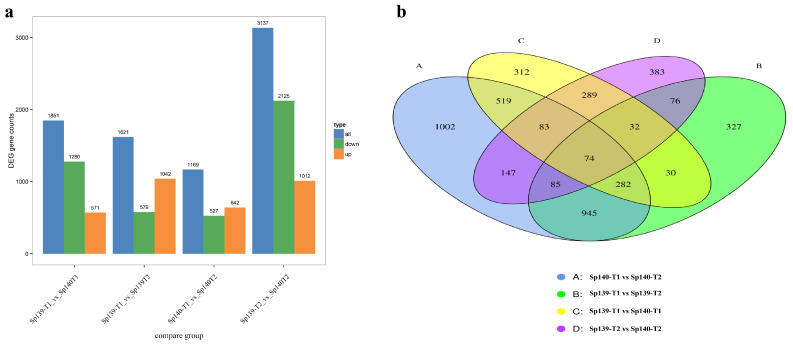
Identification of DEGs in different comparison groups. (**a**) Number of up- and downregulated DEGs in four comparisons and (**b**) Venn diagram of DEGs in four comparisons between monoecious and female plants.

The transcriptional differences between monoecious and female lines were evaluated at different stages (T1 and T2). A total of 4586 DEGs were identified across the 4 groups. In Sp139, there were 1851 DEGs (571 upregulated and 1280 downregulated) between T1 and T2. A total of 1621 DEGs (1042 upregulated and 579 downregulated) were identified in Sp140 between T1 and T2. At the T1 stage, there were 1169 DEGs (642 upregulated and 527 downregulated) between Sp139 and Sp140. At the T2 stage, 3137 DEGs (1012 upregulated and 2125 downregulated) were identified between Sp139 and Sp140 (Figure 3).

### 3.3. qRT-PCR Validation and Comparison with TPM Values

**Figure 4:**
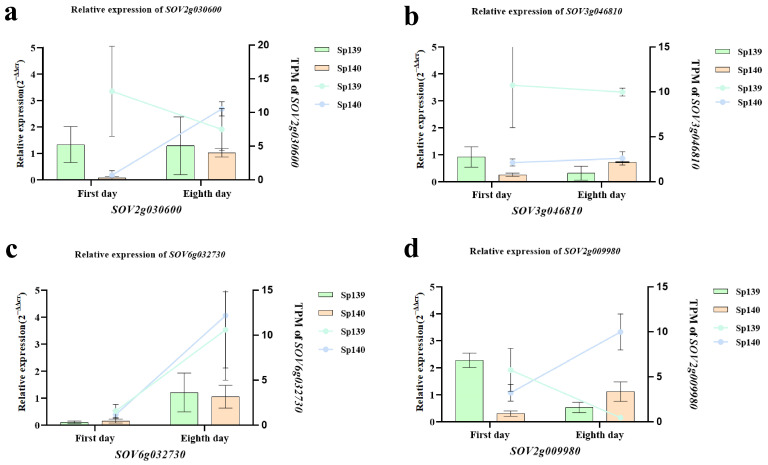
qRT-PCR was performed using four major genes. (**a**) Relative expression of *SOV2g030600*; (**b**) *SOV6g032730*; (**c**) *SOV3g046810*; and (**d**) *SOV2g009980*.

To validate the precision and reliability of RNA-seq analysis, four genes were chosen and analyzed. The results obtained from RNA-seq and qRT-PCR for these genes were highly consistent, suggesting the reliability of high-throughput transcriptome sequencing (Figure 4).

### 3.4. Enrichment Analysis for the Functional Annotation of DEGs

**Figure 5:**
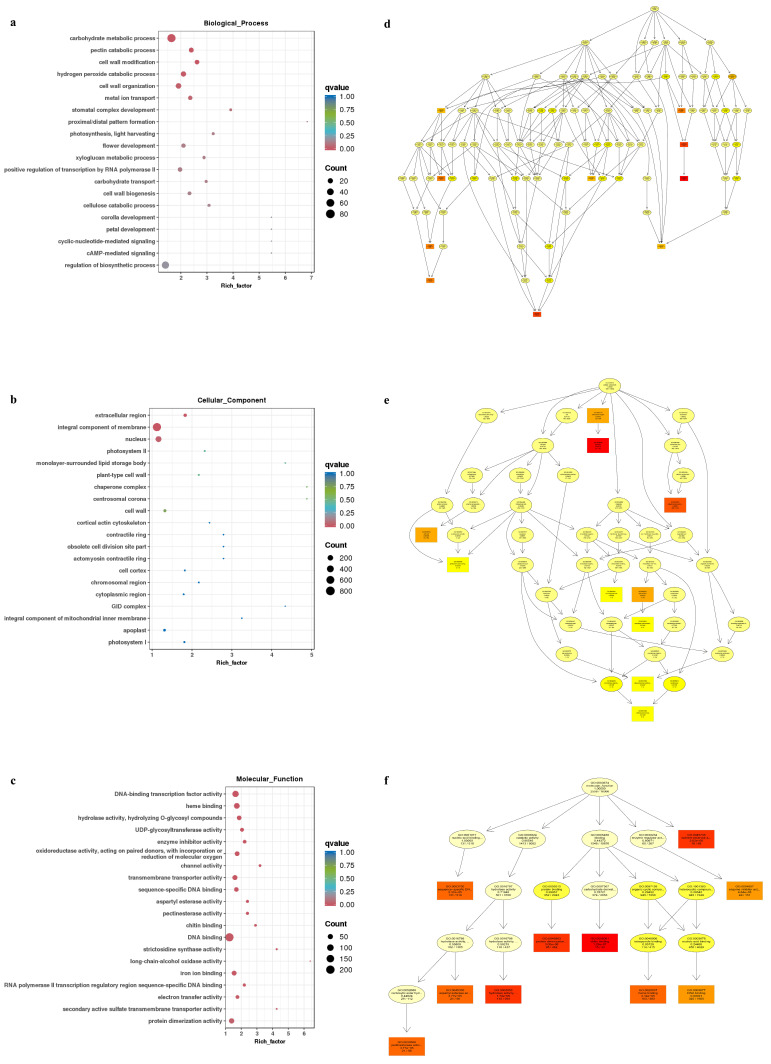
Top-20 pathways in the GO enrichment analysis of DEGs in five comparisons. (**a**) GO enrichment dot plot of Biological_Processes; (**b**) Cell_Components; (**c**) Molecular_Function; (**d**) GO terms and hierarchical relationship of Biological_Processes; (**e**) Cell_Components; and (**f**) Molecular_Function. Note: Each node represents a GO term, and the box represents the GO with an enrichment level of Top 5. The depth of the box (or ellipse) color represents the enrichment level, and the darker the color, the higher the significance. The name of the term and the q-value of the enrichment analysis are displayed on each node.

GO and KEGG analyses are used to predict the biological functions, mechanisms of action, and potential biological processes associated with genes. Enrichment analysis of the DEGs across the four groups was performed to assess their distribution in the GO and KEGG pathways. Using GO enrichment analysis, 4586 DEGs were assigned to 3 major branches: biological process, cellular component, and molecular function. Among the biological processes, the most enriched terms were related to flower and cell wall development (Figure 5a,b). In the cellular component category, the integral components of the membrane and nucleus were most significantly enriched (Figure 5c,d). DNA binding was the most enriched term in the molecular function category, indicating the regulation of gene expression and key pathways related to floral organ development and sex determination (Figure 5e,f). Among the top 20 enriched annotations, 5 were related to floral organ development or plant sex differentiation, including flower development, corolla development, and petal development (Figure 5).

**Figure 6:**
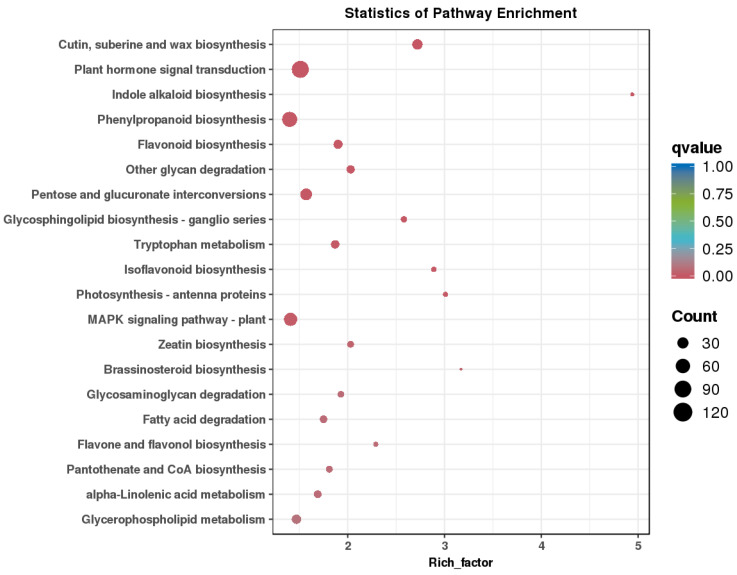
Top-20 pathways in KEGG enrichment analysis of DEGs in 5 comparisons.

In KEGG pathway analysis, the most significantly enriched pathway was indole alkaloid biosynthesis, which refers to the indole alkaloid biosynthetic process in plants. Other significantly enriched pathways with a large number of genes included plant hormone signal transduction, which regulates plant growth, development, and response to environmental stimuli, and the MAPK signaling pathway, which has a regulatory role in plant growth and development (Figure 6). Phenylpropanoid biosynthesis, which is crucial for floral organ development, was also highly enriched. Biosynthesis of zeatin and brassinosteroid (BR), which are plant hormones, were among the factors influencing sex transition in monoecious plants.

**Figure 7:**
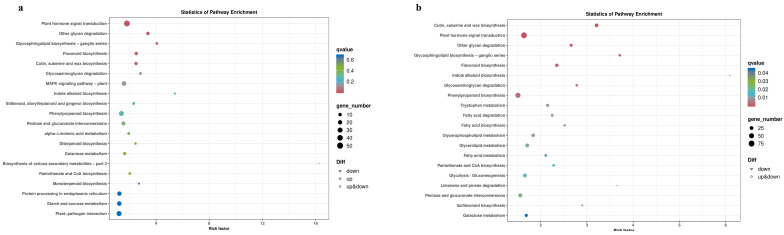
DEGs on the first and eighth day of flowering. (**a**) Top-20 pathways analyzed for KEGG enrichment of 102 DEGs in AvsB and (**b**) 113 DEGs in CvsD.

On the first day of flowering, KEGG enrichment analysis identified 102 DEGs (Figure 7a), which were significantly enriched in the pathways of plant hormone signal transduction, MAPK signaling pathway—plant, and flavonoid biosynthesis. On the eighth day of flowering, KEGG enrichment analysis revealed 113 DEGs (Figure 7b) enriched in plant hormone signal transduction and other glycan degradation pathways. These pathways play crucial roles in plant sex differentiation and flower organ development.

### 3.5. Analysis of the Co-Expression Trends of DEGs

**Figure 8:**
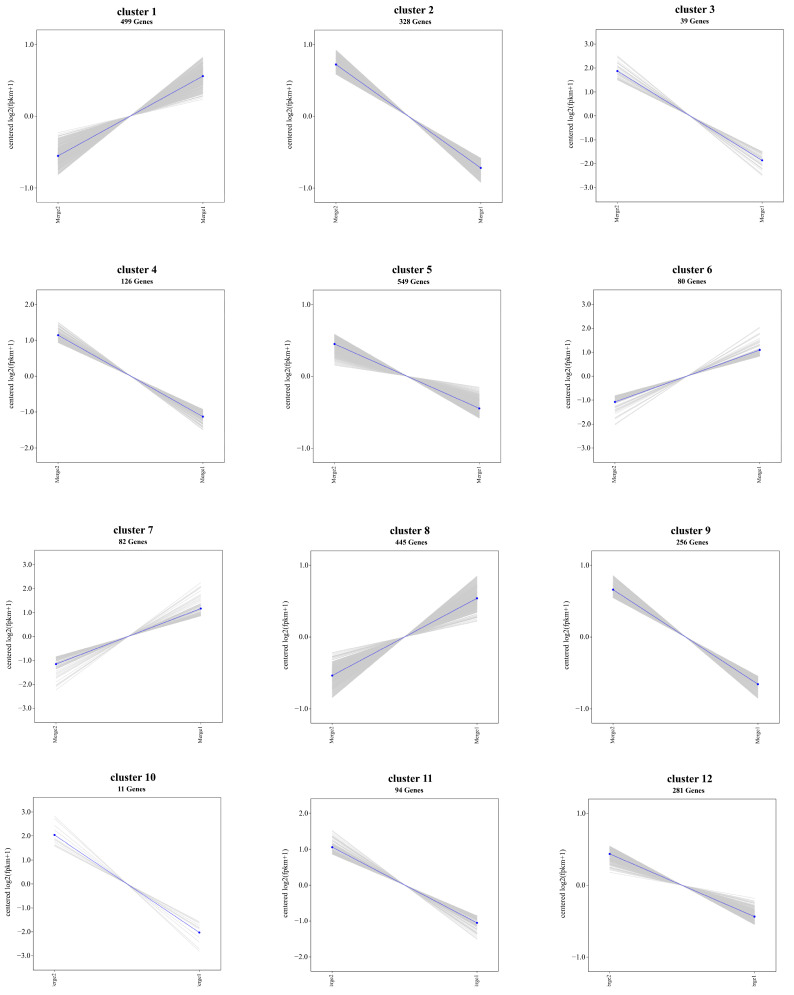
K-means clustering was used to group the expression profiles of the transcriptome into 12 clusters. Gene numbers are shown in each box. The light-gray background represents the individual expression profiles of genes within each cluster, while the blue line in the foreground depicts the overall dynamic expression trends fitted to the sample data.

Co-expression network analysis is used to identify hub genes or key regulators within each module and their interactions with other genes. A K-means clustering analysis was performed on the DEGs identified at two distinct developmental stages in monoecious plants (male and female) and female plants. Based on the dynamic expression profiles, the co-expressed genes were categorized into 6 clusters for monoecious plants and 12 clusters for female plants (Figure 8).

Among the 6 clusters for monoecious and female plants, clusters 1, 6, and 8 showed significant expression changes associated with sex determination in spinach. These clusters showed a pronounced transition from undifferentiated floral organs to differentiated structures, suggesting that the genes within these clusters play pivotal roles in the process of sex differentiation.

GO and KEGG enrichment analyses of clusters 1, 6, and 8 revealed their strong association with pathways involved in GA signaling, pollen tube development, sexual reproduction, MAPK signaling, pollen recognition, and plant hormone signaling (Supplementary Figures S1–S3). These enriched pathways were consistent with the results of enrichment analyses performed on previously identified DEGs. Together, these findings highlight that the genes involved in sex determination follow dynamic expression trends analogous to those observed in clusters 1, 6, and 8, further elucidating the molecular mechanism underlying sex differentiation in spinach.

### 3.6. Transcription Factor Prediction of DEGs

Sex differentiation in many plants is determined by the regulation of specific genes and TFs. The prediction of TFs has significant scientific and practical value in plant sex research. All 4586 DEGs were BLASTed against the Plant Transcription Factor Database (PlantTFDB) to identify the TFs. A total of 354 TFs belonging to 32 gene families were identified. Among these, 26 genes were classified as MADS-box family members.

### 3.7. Candidate Genes Associated with Monoecism

ABCDE floral identity genes play important roles in flower development and sex determination. Among the 26 MADS-box genes, 17 exhibited high homology, including 1 AP1 gene belonging to the A model, 2 PI genes belonging to the B model, and 14 AG genes belonging to the C model. Except for the MADS-box genes, six DEGs were identified as highly homologous to AP2 from the A model. In total, we uncovered 27 candidate genes related to the flower development of floral structures and organs. Nine genes (*SOV3g004950*, *SOV1g020280*, *SOV2g002560*, *SOV2g030600*, *SOV4g052720*, *SOV1g000430*, *SOV1g002920*, *SOV2g009980*, and *SOV4g045350*) were identified in the co-expression network, suggesting their roles in regulating sex differentiation between female and monoecious plants. The model genes corresponding to these 27 genes are listed in Table 1.

## 4. Discussion

Spinach is a predominantly dioecious biennial herb; however, monoecious inbred spinach lines, which have a longer flowering period and produce hybrids with high purity and yield, have significant implications for spinach breeding. Therefore, research on the monoecious form of spinach is of considerable importance for breeding programs. In this study, two sampling time points—the first day of flowering and the eighth day of flowering, which show distinct phenotypic differences—were selected for sampling two genotypes. RNA-seq analysis was performed on samples with different genotypes collected at different developmental stages. PCA, GO and KEGG enrichment analyses, co-expression trend analysis, and TF prediction were conducted based on the RNA-seq data. In this study, numerous DEGs involved in pathways related to flower development were identified, influencing processes such as floral organ morphogenesis. In addition, some DEGs were associated with plant hormone signaling pathways and phenylpropanoid biosynthesis.

GO enrichment analysis revealed that the biological processes related to corolla petal development and carbohydrate metabolic processes were the most significantly enriched pathways. Carbohydrates, a crucial organic compound in plants, play pivotal roles in plant growth, development, and metabolism. They not only serve as a primary energy source but also participate in critical physiological processes, such as cell wall biosynthesis, signal transduction, and stress responses [47]. In the context of cellular components, the chloroplast and plastid nucleoids are closely associated with chloroplast structure and function, potentially influencing plant growth and development through the regulation of photosynthesis [48]. At the molecular function level, several pathways are linked to DNA-binding activity, which is essential for cellular processes. DNA-binding activity is involved in plant hormone signaling, developmental processes, stress responses, and cell division and differentiation.

KEGG enrichment analysis revealed the most significantly enriched DEGs. Plant hormones are crucial signaling molecules regulating plant growth, development, and stress responses. Hormones, such as GAs, IAA, and BRs, are indispensable for plant growth and development [49]. These plant hormones modulate gene expression and cellular activities through intricate signal transduction pathways, thereby influencing physiological processes. In addition, the “MAPK signaling pathway—plant” is involved in a wide range of cellular processes, including growth, development, and responses to environmental stimuli.

Co-expression trend analysis identified three gene clusters whose enrichment patterns closely aligned with those observed in previous enrichment analyses. It is hypothesized that these three clusters reflect the expression trends of sex-related genes. GO and KEGG enrichment analysis of these clusters revealed that they are strongly associated with pathways such as GA signaling, nuclear development, DNA-binding, chloroplast association, sexual reproduction, and IAA response, which is consistent with the DEG enrichment results.

TFs play a crucial role in processes such as floral development, fruit ripening, and root development in plants. By predicting the TFs for all DEGs and comparing them with genes representative of the ABCDE model, 27 genes were identified. These genes regulate floral development by influencing the formation of the calyx, corolla, stamens, and carpels.

Based on functional enrichment analysis, co-expression trend analysis, and TF prediction, the *SOV3g004950* gene, encoding a MADS-box protein and showing high similarity to representative genes in the ABCDE model of floral organ development, including PI and SQUA, was enriched in the flower development pathway. The *SOV1g020280*, *SOV2g002560*, *SOV2g030600*, and *SOV4g052720* genes were significantly enriched in the DNA-binding molecular function category and exhibited high similarity to key genes in the ABCDE model, such as AP1, PI, SQUA, SEP1, and SOC1. In addition, SOV1g000430 was involved in the flower, corolla, and petal development pathways, showing high homology to genes regulating pollen development in *Arabidopsis*. The *SOV1g002920* and *SOV2g009980* genes were involved in plant hormone signaling pathways and encoded receptor-like kinases, which are highly similar to the EMS1 receptor-like kinase. These kinases are essential for plant reproductive development, particularly in microsporocyte formation, with EMS1 playing a key role in ensuring proper meiosis and anther development. The *SOV4g045350* gene belongs to the molecular function category of DNA binding and encodes a SANT domain-containing protein. This protein regulates the expression of specific genes and modulates the chromatin status, contributing to plant sex determination and floral organ development. It is a member of the RADIALIS (RAD) gene family, which influences dorsal petal identity and regulates floral symmetry. Masuda et al. [50] demonstrated that DkRAD, a gene from hexaploid persimmon, shared high homology with RADIALIS, and expression analysis showed that DkRAD likely promoted pistil development by activating MYB73 and modulating the IAA signaling pathway. Furthermore, DkRAD overexpression in *Arabidopsis* and *Nicotiana* models has been shown to induce excessive pistil growth.

We identified nine genes associated with sex determination in monoecious spinach plants. Among them, the *SOV3g004950*, *SOV1g020280*, *SOV2g002560*, *SOV2g030600*, and *SOV4g052720* genes exhibited high homology with genes involved in the floral development model. *SOV1g000430* was involved in the pathways of flower development, corolla development, and petal development, with significant expression differences across different developmental stages and genotypes. *SOV1g002920* and *SOV2g009980* influenced floral development through plant hormone signaling pathways. *SOV4g045350* was identified as a key gene involved in pistil growth.

## 5. Conclusions

The genetic study of sex determination in monoecious spinach provides a foundation for monoecious hybrid breeding and has significant implications for spinach breeding. To date, research on sex determination in spinach has been relatively limited. In this study, RNA-seq was performed using materials from monoecious and female spinach plants to analyze the functional enrichment of DEGs, co-expression trends, and TF predictions, aiming to identify key genes influencing sex determination in spinach. The results indicate that floral development and plant hormone signaling pathways are crucial for sex determination in spinach. Twenty-seven genes associated with the ABCDE model, particularly within the MADS-box and ERF families, played vital roles in spinach development. The *SOV3g004950*, *SOV1g020280*, *SOV2g002560*, *SOV2g030600*, and *SOV4g052720* genes were significantly enriched in pathways associated with floral development and exhibited high homology to key genes from the ABCDE model. *SOV1g000430* was a critical gene involved in pollen development, while *SOV1g002920* and *SOV2g009980* regulated anther structure development through plant hormones. In addition, *SOV4g045350* played a pivotal role in regulating pistil growth via the IAA signaling pathway. In conclusion, this study provided a comprehensive analysis of RNA-seq data and identified seven candidate genes associated with monoecious sex determination in spinach, laying the groundwork for future spinach breeding and sex determination research.

## Figures and Tables

**Table 1:** Gene IDs and their corresponding protein families for these hub genes.

Gene ID	The Protein Family	Similarity Represents Gene
*SOV2g002570*	MADS	AP1, PI, SQUA, SEP1, SOC1
*SOV2g002560*	MADS	AP1, PI, SQUA, SEP1, SOC1
*SOV5g027690*	MADS	AP1, PI, SQUA, SEP1, SOC1
*SOV1g019570*	MADS	AP1, PI, SQUA, SEP1, SOC1
*SOV2g030600*	MADS	AP1, PI, SQUA, SEP1, SOC1
*SOV1g020280*	MADS	AP1, PI, SQUA, SEP1, SOC1
*SOV5g010450*	MADS	AP1, PI, SQUA, SEP1, SOC1
*SOV4g032440*	MADS	AP1, PI, SQUA, SEP1, SOC1
*SOV3g038170*	MADS	AP1, PI, SQUA, SEP1, SOC1
*SOV3g046800*	MADS	AP1, PI, SQUA, SEP1, SOC1
*SOV4g008150*	MADS	AP1, PI, SQUA, SEP1, SOC1
*SOV4g052950*	MADS	AP1, PI, SQUA, SEP1, SOC1
*SOV3g046460*	MADS	AP1, PI, SQUA, SEP1, SOC1
*SOV3g046810*	MADS	AP1, PI, SQUA, SEP1, SOC1
*SOV4g052720*	MADS	AP1, PI, SQUA, SEP1, SOC1
*SOV3g018900*	MADS	AP1, PI, SQUA, SEP1, SOC1
*SOV5g010460*	MADS	AP1, PI, SQUA, SEP1, SOC1
*SOV4g059500*	MADS	AP1
*SOV1g020300*	MADS	PI, SQUA
*SOV1g047710*	MADS	PI, SQUA
*SOV3g004950*	MADS	PI, SQUA
*SOV6g045620*	ERF	AP2
*SOV6g045610*	ERF	AP2
*SOV6g032730*	ERF	AP2
*SOV3g042190*	ERF	AP2
*SOV3g004160*	ERF	AP2
*SOV1g045680*	ERF	AP2

## Data Availability

The transcriptomic data has been uploaded to the National Genomics Data Center (NGDC) and can be accessed using the BioProject accession number PRJCA033895 at the following URL: https://ngdc.cncb.ac.cn/ (accessed on 23 December 2024).

## Supplementary Materials

The following supporting information can be downloaded at: https://www.mdpi.com/article/10.3390/genes16010024/s1, Table S1: Summary of Illumina RNA-seq data; Table S2: Primer sequences for qRT-PCR; Figure S1: Top 20 pathways in the GO and KEGG enrichment analysis of DEGs in Cluster 1; Figure S2: Top 20 pathways in the GO and KEGG enrichment analysis of DEGs in Cluster 6; Figure S3: Top 20 pathways in the GO and KEGG enrichment analysis of DEGs in Cluster 8.
