# Supplementary figures and images for: Comparative Transcriptome Analysis of Gene Expression Between Female and Monoecious Spinacia oleracea L
Source: Genes (Basel). 2024 Dec 27;16(1):24. doi: 10.3390/genes16010024 (PMC11764767; doi:10.3390/genes16010024)

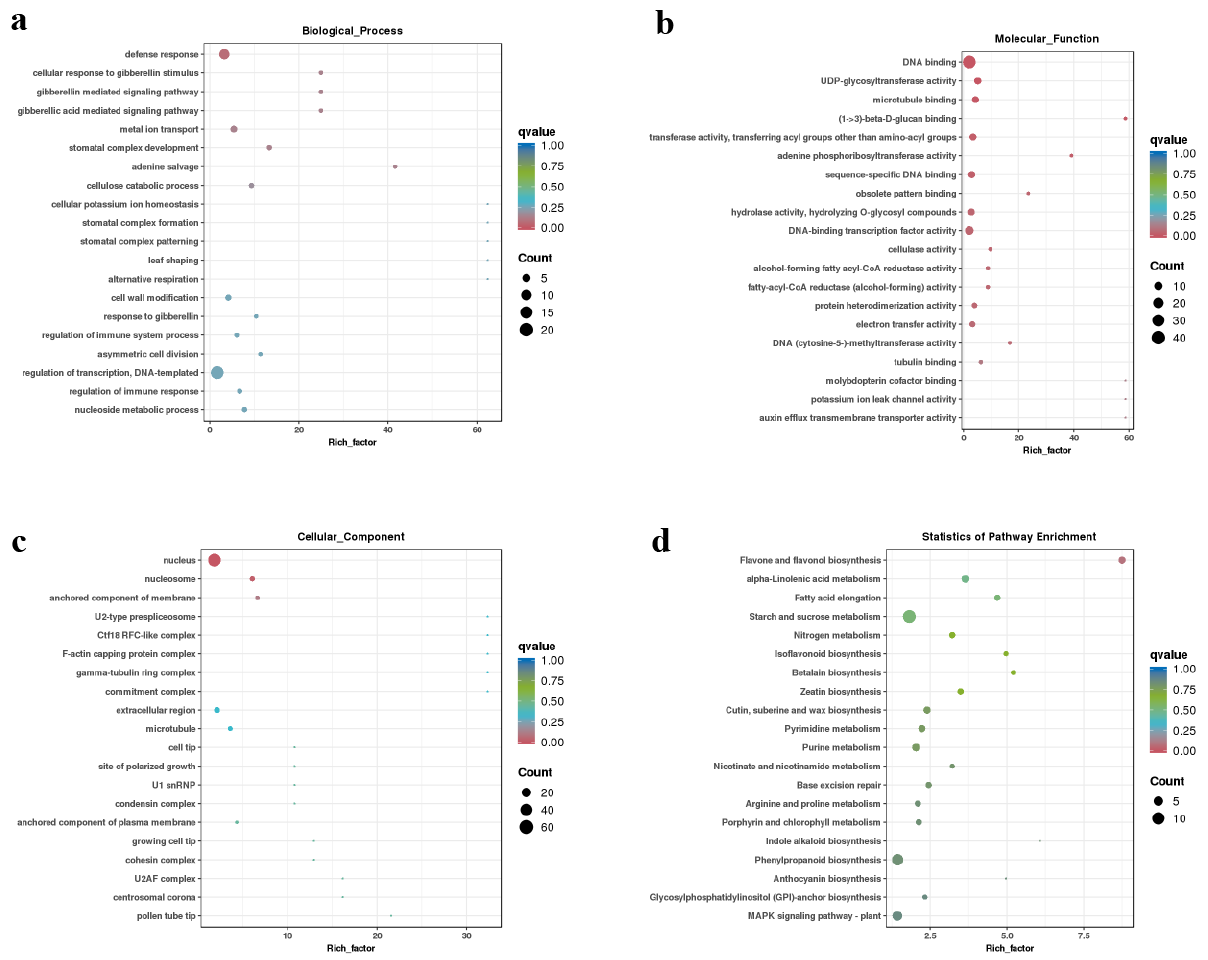

Supplement: Supplementary file 1 [file genes-16-00024-s001.zip › Figure S1.png]

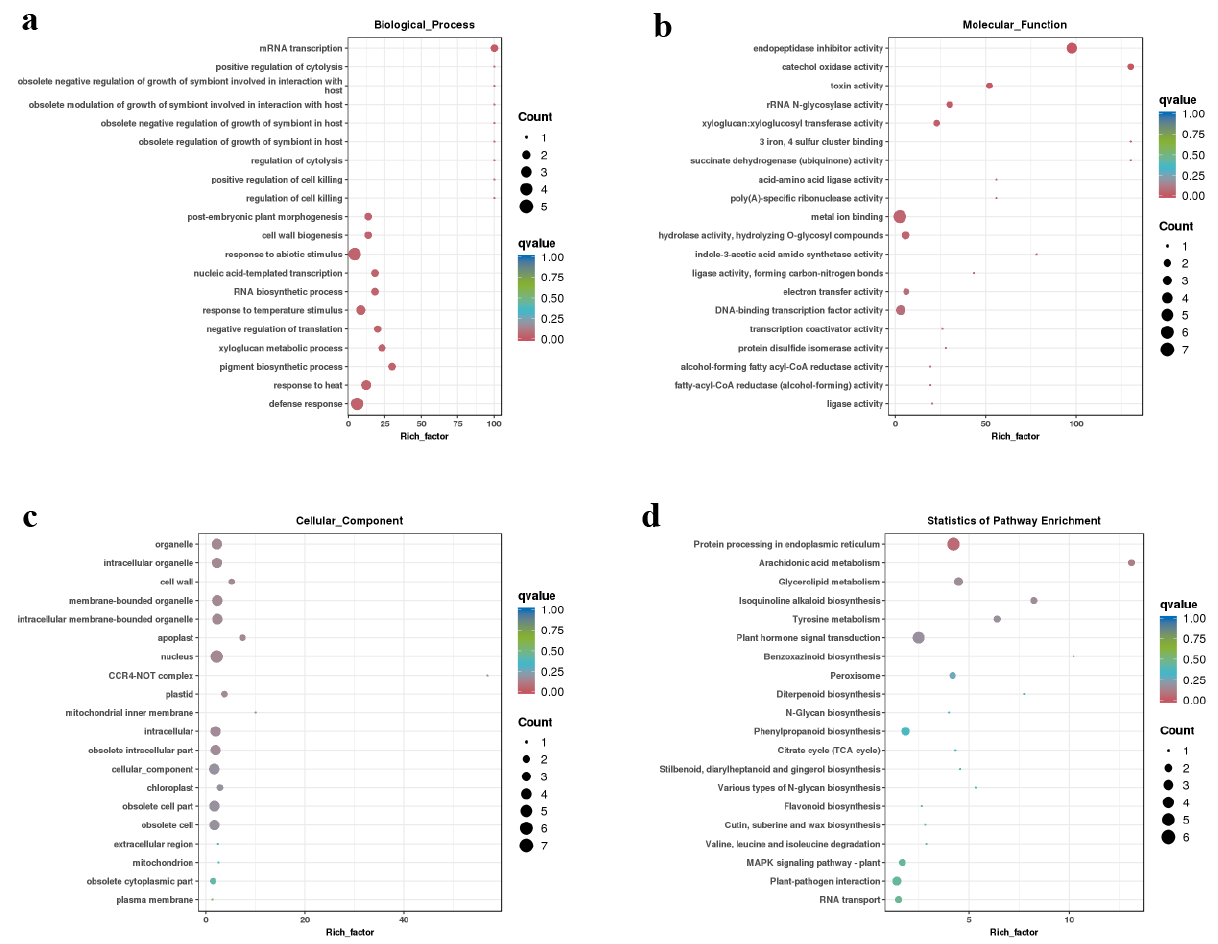

Supplement: Supplementary file 1 [file genes-16-00024-s001.zip › Figure S2.png]

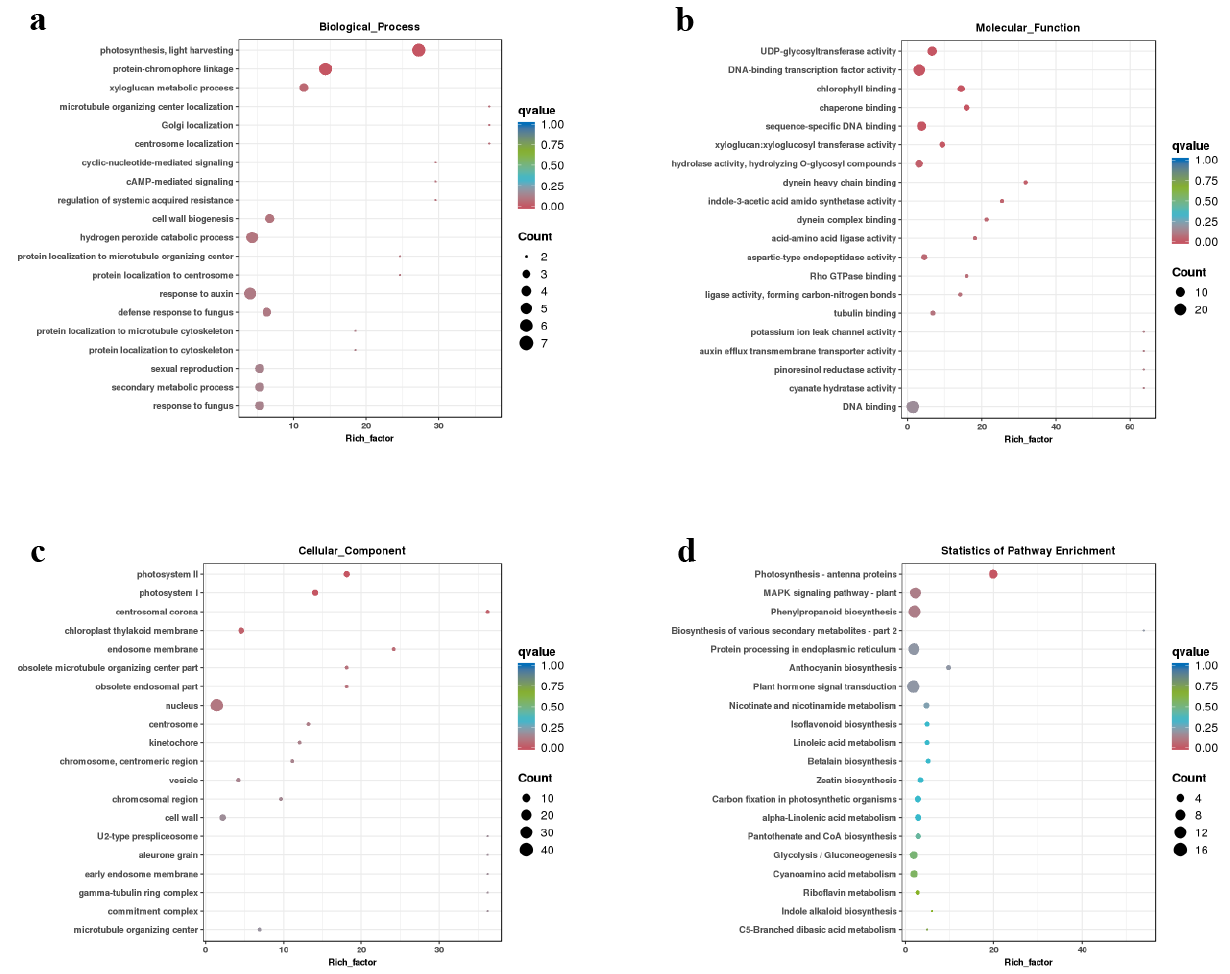

Supplement: Supplementary file 1 [file genes-16-00024-s001.zip › Figure S3.png]
